# Enhanced image fusion using directional contrast rules in fuzzy transform domain

**DOI:** 10.1186/s40064-016-3511-8

**Published:** 2016-10-22

**Authors:** Amita Nandal, Hamurabi Gamboa Rosales

**Affiliations:** 1 Electronics and Communication Engineering Department, National Institute of Technology, Hamirpur, Himachal Pradesh India; 2 Faculty of Computers and Electrical Engineering, University of Science and Information Technology, Partizanska, Ohrid, 6000 Republic of Macedonia; 3 Faculty of Electrical Engineering, Autonomous University of Zacatecas, Zacatecas, Mexico

**Keywords:** Directional contrast, Image fusion, Fuzzy transform, Quantitative fusion metrics

## Abstract

In this paper a novel image fusion algorithm based on directional contrast in fuzzy transform (FTR) domain is proposed. Input images to be fused are first divided into several non-overlapping blocks. The components of these sub-blocks are fused using directional contrast based fuzzy fusion rule in FTR domain. The fused sub-blocks are then transformed into original size blocks using inverse-FTR. Further, these inverse transformed blocks are fused according to select maximum based fusion rule for reconstructing the final fused image. The proposed fusion algorithm is both visually and quantitatively compared with other standard and recent fusion algorithms. Experimental results demonstrate that the proposed method generates better results than the other methods.

## Background

Image fusion is the process of fusing different images to increase the amount of significant information. These images are obtained either from different imaging modalities or from single modality. Different image fusion methods have been developed in literature (James and Dasarathy 2014). Recently, fuzzy logic based image fusion methods (Seng et al. 2010; Kayani et al. 2007) are gaining interest of researchers. Fuzzy logic has revealed to provide a basis for the approximate description of different functions. Motivated from fuzzy logic and system modeling, Perfilieva (2006) introduced fuzzy-transform (FTR/F-transform) that maps a set of functions in one space into a finite dimensional vector in another space. Researchers have successfully applied FTR in many applications including image compression, image fusion, image denoising, time series application etc. Perfilieva and Dankova (2008) proposed simple FTR based image fusion algorithm based on one-level decomposition and complete F-transform based image fusion algorithm based on high level decomposition. Maximum absolute value corresponding to least degraded part of input image was used as an operator for performing fusion. However, these algorithms could not be successfully applied to fuse images. The fused image obtained was of poor contrast as the FTR components corresponding to the smoother part in the image was not within the range of fusion operator. So, to obtain a fused image of good contrast, Perfilieva et al. modified original images by enhancing their contrast and then applied the proposed algorithm on newly obtained input images. This paper proposes image fusion method that fuses original images as such where directional contrast is enhanced using FTR. The rest of the paper is organized as follows: Second section gives literature review, third section presents the proposed method, results are given and discussed in fourth section and finally, conclusion is drawn in fifth section.

## Literature review

The main aim of image fusion methods (Piella 2003) is to preserve all salient, interrelated and relevant information present in input images without introducing any inconsistency, noise and artifact in the fused image. An important requirement for successful fusion of input images is to have accurate geometric alignment that requires proper matching of image coordinates. This can be achieved through a process known as image registration (Bhattacharya and Das 2011). Commonly used spatial domain pixel-level algorithms (Zoran 2009) include averaging based, select maxima or minima based, intensity hue saturation (IHS) transform based, principal component analysis (PCA) based, Brovey transform (BT) based fusion methods. In the transform-domain, first the input images are transformed into frequency domain and then fusion takes place according to some fusion rules in transform domain. Finally inverse transform is done to achieve a final fused image. Guihong et al. (2001) proposed modulus maxima value of the wavelet coefficients at each point as a fusion rule to produce a fused image. Modulus maxima based fusion rule extracts sharp signal transitions and singularity features but is also sensitive to noise and artifacts. Some authors also proposed image fusion based on multiwavelet transform that possess many desirable properties such as orthogonality, symmetry and smoothness. Liu et al. (2010) proposed that either gradient based or weighted average based fusion method can be used for determining the fused low frequency coefficients where, either an algorithm based on maximum value or directional contrast or classification scheme can be used for determining the fused high frequency coefficients. On the other hand, Tongzhou et al. (2009) proposed a feature-based fusion rule to fuse original sub-images. Combination of four different fusion rules: average, addition, principal component selection and select maxima were used to fuse the coefficients of low frequency sub-band and high frequency sub-band. Since directional contrast using FTR has good approximation properties and is successful in preserving true image edges, researchers (Vajgl et al. 2012; Dankova and Valăsek 2006) have also proposed fusion of multiple images using fuzzy transform. Motivated from these properties and various advantages of FTR this paper proposes fusion based on FTR domain with directional contrast.

FTR, introduced by Perfilieva (2005, 2006), is a powerful transformation technique that is capable of preserving features especially for fuzzy models. It has been successfully applied to a wide range of applications such as image fusion (Perfilieva and Dankova 2008; Vajgl et al. 2012; Dankova and Valăsek 2006), image compression (Perfilieva and Baets 2010; Martino et al. 2008), noise removal, data analysis, solution of differential and integral equations (Ezzati and Mokhtari 2012) etc. FTR establishes a correspondence between a set of functions in a closed interval into a finite (say *N*) dimensional vector space. It has an advantage of producing a simple and unique representation of an original function when used in place of original function and it makes complex computations easier. FTR is as useful as traditional transforms such as wavelet transform and Fourier transform, but FTR has a potential advantage over these transforms as it can use several basis functions of different shapes whereas wavelet transform utilizes a single mother wavelet to define all basis functions and Fourier transform uses only a single kind of basis function i.e. *e* ^*jwx*^ (Patanè 2011).

The performance of image fusion algorithms is usually bounded by two factors: the algorithm quality and the quality of the registration results (He et al. 2010). A multimodal image registration and fusion module (MIRF) is proposed in (Ghantous and Bayoumi 2013). MIRF is able to automatically register and fuse images with the use of multi-resolution decomposition based on Dual-Tree Complex Wavelet Transform (DT-CWT). An important requirement for successful fusion of input images is to have accurate geometric alignment that requires proper matching of image coordinates (Petrovic and Xydeas 2005).

The performance and visual quality of image is retained using discrete cosine harmonic wavelet (DCHWT) based image fusion with reduced computation (Kumar 2013). A fused image with maximum number of measures achieving their desirable value is considered to be a better quality of fused image. Many objective measures have been developed in literature for assessing the performance of image fusion algorithms. The measures generally used for evaluating the performance of fusion algorithms are based on the amount of information that has been transferred from the input images into fused image (Kotwal and Chaudhuri 2013; Haghighat et al. 2011; Arathi and Soman 2009; Wang et al. 2004; Zhang et al. 2011; Liu and Laganiere 2007).

Multilevel Dual-Tree Complex Wavelet Transform (DT-WT) is also a comparable method but it requires the design of special filters with desirable properties: approximate half-sample delay property, perfect reconstruction (orthogonal or bi-orthogonal), finite support, vanishing moments (good stop band) and linear phase characteristics. Also since DT-WT involves complex coefficients, processing these (both real and imaginary) coefficients increases the computational complexity and the memory requirement, thereby increasing the cost of fusion method (Singh and Khare 2014).

A fusion framework is proposed for multimodal medical images based on non-subsampled contourlet transform (NSCT) (Wang et al. 2013), based on which we can represent low-frequency information of the image sparsely in order to extract the salient features of images. Furthermore, it can reduce the calculation cost of the fusion algorithm with sparse representation by the way of non-overlapping blocking, thereby increasing complexity of fusion.

The shift-invariant shearlet transform (SIST) method can efficiently capture both of the spatial feature information and the functional information contents. Besides, different from the average and maximum schemes the dependencies of the SIST coefficients of the cross-scale and inter sub-bands have been fully considered in the proposed fusion rule, and therefore more information from the source images can be transferred into the fused images (Wang et al. 2014).

The contrast feature measures the difference of the intensity value at some pixel from the neighbouring pixels which is presented as directive contrast in NSCT domain method (Bhatnagar et al. 2013). For fusion, two different rules are used by which more information can be preserved in the fused image with improved quality. That is why, in our proposed method images are fused according to directional contrast based fusion and select maximum based fusion. The proposed fusion algorithm is also compared subjectively as well as objectively with MIRF (Ghantous and Bayoumi 2013), DCHWT (Kumar 2013), Multilevel DT-WT (Singh and Khare 2014), NSCT (Wang et al. 2013), SIST (Wang et al. 2014) and Directive Contrast in NSCT (Bhatnagar et al. 2013).

## Proposed method

**Fig. 1:**
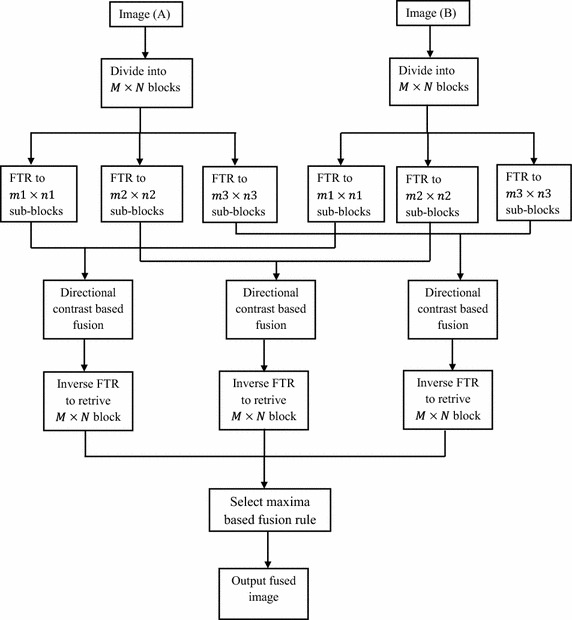
Block diagram of proposed algorithm

**Fig. 2:**
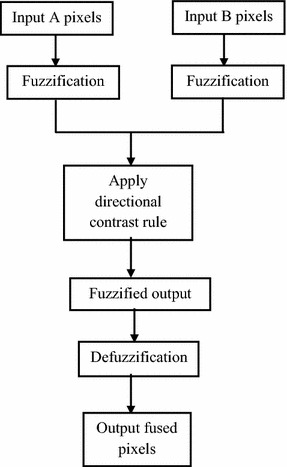
Block diagram for fuzzy transformation and defuzzification

Selection of proper fusion rules should be carefully made in order to provide a better quality of fused image. In this work directional contrast rule in fuzzy transform (FTR) domain is proposed. Contrast enhancement is based on emphasizing the difference of brightness in an image to improve its perceptual quality (Gonzalez and Woods 2002; Peli 1990). The spatial content is equally important for defining the contrast. Using this property we have considered two bands of frequency one is high another is low, where each frequency band is a function of the contrast. We define metrics to measure the contrast enhancement, and luminance/brightness to measure the image quality of the contrast-enhanced images. The proposed method is based on fusion of two different tone images. This is achieved using fuzzy technique which is described in paper (Hanmandlu et al. 2003). The block diagram of proposed method is illustrated in Fig. 1. The block diagram for fuzzy transformation and defuzzification is presented in Fig. 2.

The performance of the proposed method is evaluated using quantitative measures and subjective perceptual image quality evaluation. So high and low frequency components in a (2*w*1 + 1) × (2*w*2 + 1) window is calculated and the values with maximum and minimum contrast is chosen as the fused transformed component. Here *w*1 and *w*2 being positive integer. The use of maximum and minimum contrast is used to find out normalized value which is a part of proposed algorithm. The contrast of an image can be defined as,

1 $$R = \frac{{L - L_{B} }}{{L_{B} }} = \frac{{L_{H} }}{{L_{B} }}$$

where, R is the contrast of the image, L is the local grey level, L_B_ is the local brightness of the background (corresponding to low frequency component), L_H_ = L − L_B_ corresponds to high frequency component. After one level of wavelet decomposition, there will be four frequency bands, namely three high frequency components *D*_*i*,*j*_^*H*^, *D*_*i*,*j*_^*V*^ and *D*_*i*,*j*_^*D*^ (corresponding to the “foreground” i.e. Horizontal, vertical and Diagonal) and one low frequency component *C*_*i,j*_ (corresponding to the “background”). The value of *i* and *j* depends on the block of images. The exact relationship is explained in proposed algorithm. Because of orthogonally of decomposition, there isn’t relativity between the high frequency component (“foreground”) and the low frequency component (“background”), and so the improvement seen using directional contrast is reasonable.

### Proposed algorithm

Input images *X* and *Y* are initially divided into blocks of size *M* × *N*. Since images generally contain different types of spatial degradation, that disrupts its smoothness, hence each *M* × *N* block of both images is fuzzy transformed into sub-blocks (SB) of size (*m*1 × *n*1), (*m*2 × *n*2) and (*m*3 × *n*3) using FTR. Fusion is performed for each block, performing following steps. Assume *SB*_*X*_ referes to subblock of image X and *SB*_*Y*_ refers to sub-block of image Y. DWT is applied to these sub-blocks. After one level of wavelet decomposition, there will be four frequency bands, namely three high frequency components *D*_*i*,*j*_^*H*^, *D*_*i*,*j*_^*V*^ and *D*_*i*,*j*_^*D*^ (corresponding to the “foreground” i.e. Horizontal, vertical and Diagonal) and one low frequency component *C*_*j*_ (corresponding to the “background”). The components of fused image is represented as, *SB*_*Fused*_ → [*C*_*i*,*j*_^*F*^, *D*_*i*,*j*_^*HF*^, *D*_*i*,*j*_^*VF*^, *D*_*i*,*j*_^*DF*^]. The notation *F* refers to fused image. *C*_*j*_^*F*^ refers to low frequency component of fused image. *D*_*j*_^*HF*^, *D*_*j*_^*V*^, *D*_*j*_^*DF*^ referes to high frequency component of fused image corresponding to horizontal, vertical and diagonal directional metrics respectively.

2 $$C_{i,j}^{F} = \frac{{C_{j}^{{SB_{X} }} + C_{j}^{{SB_{Y} }} }}{2}$$

2a $$D_{i,j}^{HF} = FTA \left(D_{i,j}^{{H_{{SB_{{l_{X} }} }} }} ,D_{i,j}^{{H_{{SB_{{l_{Y} }} }} }} \right)$$

2b $$D_{i,j}^{VF} = FTA \left(D_{i,j}^{{V_{{SB_{{l_{X} }} }} }} ,D_{i,j}^{{V_{{SB_{{l_{Y} }} }} }} \right)$$

2c $$D_{i,j}^{DF} = FTA \left(D_{i,j}^{{D_{{SB_{{l_{X} }} }} }} ,D_{i,j}^{{D_{{SB_{{l_{Y} }} }} }} \right)$$

- *i* = 1, 2, …, *m*1, *j* = 1, 2, …, *n*1 and *l* = *m*1 × *n*1 for subblocks of size *m*1 × *n*1.
- *i* = 1, 2, …, *m*2, *j* = 1, 2, …, *n*2 and *l* = *m*2 × *n*2 for subblocks of size *m*2 × *n*2
- *i* = 1, 2, …, *m*3, *j* = 1, 2, …, *n*3 and *l* = *m*3 × *n*3 for subblocks of size *m*3 × *n*3

*l* is sub-block of input images *A* and *B* and *i*, *j* specify location. The window is centered at location *i*, *j*.

In the proposed technique, fuzzy intensification is suggested on the basis of optimization of directional contrast using fuzzy transformation. A Gaussian membership function that transforms the saturation and intensity histograms of HSV colour model. The fuzzifier and intensification parameters are evaluated automatically for the input colour image by optimizing the contrast in the fuzzy domain. It is observed that the “index of fuzziness” decrease with enhancement. It has been found that RGB colour model is not suitable for enhancement because the colour components are not decoupled. On the other hand, in HSV colour model, hue (H), the colour content is separated from saturation (S), which can be used to dilute the colour content and V, the intensity (Value) of the colour content. By preserving H, and changing only S and V, it is possible to enhance colour image (Ezzati and Mokhtari 2012; Kumar 2013). Therefore, we need to convert RGB into HSV for the purpose. A Gaussian type membership function is used to model S and V property of the image. This membership function uses only one fuzzifier and is evaluated by maximizing fuzzy contrast, which is cumulative fuzzy variance about the crossover point. The colour image is first converted from RGB to HSV domain to preserve the hue of the image. We have considered an image set i.e., Room for the demonstration. A clear improvement is seen as far as the details and restorations of colours are concerned.

#### Fuzzy transformation algorithm

- *Step 1*: Calculate normalized value of each input pixel contrast using

7 $$x_{norm} \left( {i,j} \right) = \frac{{(x\left( {i,j} \right) - x_{min} )}}{{(x_{max} - x_{min} )}}$$

*x*_*max*_ and *x*_*min*_ are maximum and minimum contrast of pixel in each block.

$x\left( {i,j} \right) = \frac{1}{{C _{i,j} }}\sqrt {D_{i,j}^{H2} + D_{i,j}^{V2} + D_{i,j}^{D2} }$, where *x*(*i*, *j*) is absolute value of the image gradient taken as a simple indicator of the image contrast. Contrast enhancement (CE) operator cab be represented as, $CE = \frac{1}{i \times j}\left( {\mathop \sum \nolimits_{i} \mathop \sum \nolimits_{j} D} \right)$.

- *Step 2*: Calculate the crossover membership value of each block using

8 $$\mu_{crossover} = \frac{{(1 + \frac{{x_{min} }}{{x_{max} }})}}{{2(1 - \frac{{x_{min} }}{{x_{max} }})}}$$

- *Step 3*: Fuzzify the image using the following steps

If $$0 < x_{norm(i,j)} < \mu_{crossover} ,$$ then

9 $$f_{img} \left( {i,j} \right) = \frac{{x_{norm}^{{2^{r} }} (i,j)}}{{(1 - \mu_{crossover} )^{{2^{(r - 1)} }} }}$$

where *r* is radius of Gaussian membership function and $f_{img} \left( {i,j} \right)$ is final fuzzified image.

else, $$\mu_{crossover} \_ x_{norm(i,j)} < 1$$, and

10 $$f_{img} \left( {i,j} \right) = 1 - \frac{{x_{norm}^{{2^{r} }} \left( {i,j} \right)}}{{\left( {1 - \mu_{crossover} } \right)^{{2^{{\left( {r - 1} \right)}} }} }}$$

- *Step 4*: The pixel intensity of defuzzified image (*df*_*img*_(*i*, *j*)) is obtained using,

11 $$df_{img} \left( {i,j} \right) = f_{img} \left( {i,j} \right) \times \left( {x_{max} - x_{min} } \right)$$

#### Select maximum fusion rule

These fused transformed sub-blocks are then inverse transformed into original size blocks using inverse FTR. These inverse FTR blocks are further fused using select maximum based fusion rule for producing a final fused block of size *M* × *N*. After enhancing the images using directional contrast, they are fused using discrete wavelet transform (DWT) for extracting various features of the images at different levels. Select maximum fusion rule is applied as follows,

- *Step 1*: Obtain sub-block decomposition of both images.
- *Step 2*: Apply fusion rule as

12 $$F_{M \times N} \left( {i,j} \right) = \left\{ {\begin{array}{*{20}c} {InvF^{{SB_{m1 \times n1} \left( {i,j} \right) }} ,\quad {\text{if}}\quad InvF^{{SB_{m1 \times n1} \left( {i,j} \right) }} \ge (InvF^{{SB_{m2 \times n2} \left( {i,j} \right)}} \;{\text{and}}\quad InvF^{{SB_{m3 \times n3} (i,j)}} )} \\ {InvF^{{SB_{m2 \times n2} (i,j)}} ,\quad {\text{if}}\quad InvF^{{SB_{m2 \times n2} (i,j)}} \ge (InvF^{{SB_{m1 \times n1} \left( {i,j} \right) }} \;{\text{and}}\quad InvF^{{SB_{m3 \times n3} (i,j)}} )} \\ {InvF^{{SB_{m3 \times n3} (i,j)}} ,\quad {\text{if}}\quad InvF^{{SB_{m3 \times n3} (i,j)}} \ge (InvF^{{SB_{m1 \times n1} \left( {i,j} \right) }} \;{\text{and}}\quad InvF^{{SB_{m2 \times n2} (i,j)}} )} \\ \end{array} } \right.$$

where $InvF^{{SB_{m1 \times n1} \left( {i,j} \right)}}$ represents inverse FTR of subblock of size $m1 \times n1.$ This maximum value of inverse FTR ensures that the dominant features of input images are incorporated as completely as possible in the final fused image.

#### Inverse FTR

The Inverse FTR is calculated using DWT coefficients for extracting various features of the images at different levels by choosing the *I*_*Xcoef*_ and $I_{Ycoef }$. $I_{Xcoef}$ and $I_{Ycoef }$ are the DWT coefficients of Image *X* and Image *Y* images respectively.

Apply fusion rule as,

13 $$fused_{coef} = \left\{ {\begin{array}{ll} {I_{Xcoef} } & {if \quad I_{Xcoef} > I_{Ycoef} } \\ {I_{Ycoef} } & {otherwise} \\ \end{array} } \right.$$

DWT increases directional information and introduces no blocking artifacts, thereby providing better perceptual image quality. Finally, take the inverse DWT of fused image coefficients to obtain the final fused image. Reconstruct the image, F using these fused *F*_*M*×*N*_ blocks.

## Results and discussions

**Fig. 3:**
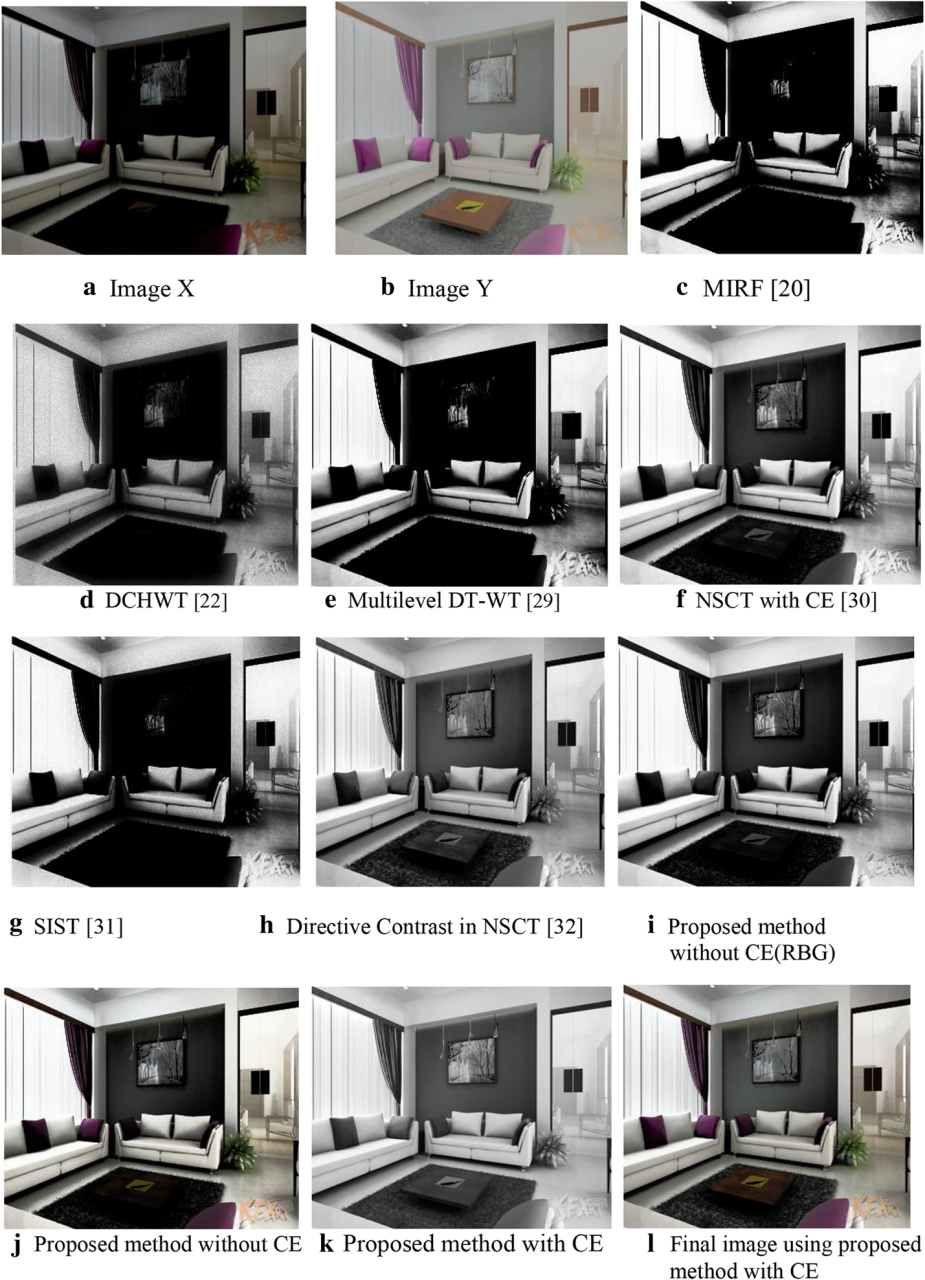
Qualitative result from different image fusion methods

**Fig. 4:**
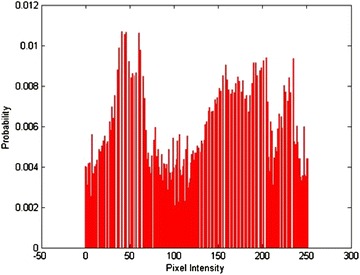
Pixel intensity without CE

**Fig. 5:**
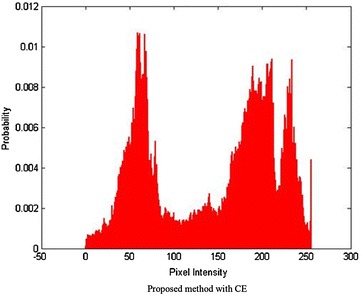
Pixel Intensity with increased contrast

Different images are fused to evaluate the performance of the proposed algorithm. The fusion algorithm is performed by decomposing input images into non-overlapping blocks of size 8 × 8 and then fuzzy transforming them into sub blocks of size 3 × 3, 5 × 5 and 7 × 7. From these results it is observed that small size sub-blocks are at coarser resolution level, representing approximation information such as texture of input images whereas larger size sub-blocks are at high resolution level, containing detail information such as edges and boundaries of input images. However the proposed method is successful in fusing the approximation as well as the finer details of input images in the fused image. Experimentally, it has been found that a 3 × 3 window size is more effective in terms of their entropy values reported. Since FTR has the capability of preserving monotonicity and Lipschitz continuity (Perfilieva and Baets 2010) of a function/true image edges, the proposed fusion method provides better fusion results. Figure 3 shows the qualitative comparison of various fusion methods. Visual results indicate that the proposed algorithm produces a better quality of fused image with important information well preserved in the resultant image. Figures 4 and 5 show histogram for pixel intensity without contrast enhancement and with contrast enhancement.

### Performance measures

Performance measures such as: edge strength(*Q*_*XY*_^*F*^) proposed by Petrovic and Xydeas (2005), fusion loss(*FL*_*XY*_^*F*^) (Kumar 2013), fusion artifacts (*FA*_*XY*_^*F*^) (Kotwal and Chaudhuri 2013), entropy (*H*_*XY*_^*F*^) (Haghighat et al. 2011), standard deviation (*SD*_*XY*_^*F*^) (Haghighat et al. 2011), feature mutual information (*FMI*_*XY*_^*F*^) (Arathi and Soman 2009), fusion factor (*FF*_*XY*_^*F*^) (Wang et al. 2004), fusion symmetry (*FS*_*XY*_^*F*^) (Wang et al. 2004), structural similarity index measure (*SSIM*_*XY*_) (Zhang et al. 2011) and feature similarity index measure (*FSIM*_*XY*_^*F*^) (Liu and Laganiere 2007) are widely used in evaluating the performance of fusion methods. A fused image with maximum number of measures achieving their desirable value is considered to be a better quality of fused image. Many objective measures have been developed in literature for assessing the performance of image fusion algorithms but none of the measure has been considered as a standard measure. The main reason of not defining a proper quality measure for image fusion is the difficulty in defining an ideal fused image. The measures generally used for evaluating the performance of fusion algorithms are based on the amount of information that has been transferred from the input images into fused image.

#### Edge strength

Edge strength (*Q*_*XY*_^*F*^) measure, proposed by Petrovic and Xydeas (2005), determines the relative amount of edge information that is transferred from the input images into the fused image. It is determined by using the edge preservation values *Q*_*XF*_(*i*, *j*) and *Q*_*YF*_(*i*, *j*) for image X and Y and is calculated as:

14 $$Q_{XY}^{F} = \frac{{\mathop \sum \nolimits_{i = 1}^{M} \mathop \sum \nolimits_{j = 1}^{N} [Q_{XF} \left( {i,j} \right)w_{X} \left( {i,j} \right) + Q_{YF} \left( {i,j} \right)w_{Y} \left( {i,j} \right)]}}{{\mathop \sum \nolimits_{i = 1}^{M} \mathop \sum \nolimits_{j = 1}^{N} [w_{X} \left( {i,j} \right) + w_{Y} \left( {i,j} \right)]}}$$

where *w*_*X*_(*i*, *j*) and *w*_*Y*_(*i*, *j*) are the weights assigned to *Q*_*XF*_(*i*, *j*) and *Q*_*YF*_(*i*, *j*) respectively. The large value of *Q*_*XF*_(*i*,*j*) depicts better edge information in the fused image.

#### Fusion loss

In practice, not all of the information present in the input images is transferred into the fused image. Some information of the input images get necessarily lost in the fusion process. This loss of input information is obtained as a perceptual weighted summation of local fusion loss, defined as (1 − *Q*_*XF*_(*i*, *j*)) and (1 − *Q*_*YF*_(*i*, *j*)) for images X and Y respectively, over locations where gradient strength in the fused image is weaker than its value in the input images. Mathematically, fusion loss (*FL*_*XY*_^*F*^) (Kumar 2013) is defined as:

15 $$FL_{XY}^{F} = \frac{{\mathop \sum \nolimits_{i = 1}^{M} \mathop \sum \nolimits_{j = 1}^{N} p(i,j)[(1 - Q_{XF} \left( {i,j} \right))w_{X} \left( {i,j} \right) + (1 - Q_{YF} \left( {i,j} \right))w_{Y} \left( {i,j} \right)]}}{{\mathop \sum \nolimits_{i = 1}^{M} \mathop \sum \nolimits_{j = 1}^{N} [w_{X} \left( {i,j} \right) + w_{Y} \left( {i,j} \right)]}}$$

where,

$$p\left( {i,j} \right) = \left\{ {\begin{array}{ll} {1, \quad if\,s_{F} \left( {i,j} \right) < s_{X} \left( {i,j} \right) or \, s_{F} \left( {i,j} \right) < s_{Y} \left( {i,j} \right)} \\ {0, \quad else} \\ \end{array} } \right.$$

where *s*_*X*_(*i*, *j*), *s*_*Y*_(*i*, *j*) and *s*_*F*_(*i*, *j*) represents (information about) gradient strength at location $(i, j)$ in $X, Y$ and $F$ respectively.

#### Fusion artifacts

Many times fusion process itself creates unwanted artifacts in the fused image, which may affect the performance of certain fusion applications. These artifacts are obtained as a perceptual weighted summation of fusion noise at locations where fused gradient strength is stronger than that of its value in both the input images. Mathematically, fusion artifacts (Kotwal and Chaudhuri 2013) are calculated as:

16 $$FA_{XY}^{F} = \frac{{\mathop \sum \nolimits_{i = 1}^{M} \mathop \sum \nolimits_{j = 1}^{N} q(i,j)[(1 - Q_{XF} \left( {i,j} \right))w_{X} \left( {i,j} \right) + (1 - Q_{YF} \left( {i,j} \right))w_{Y} \left( {i,j} \right)]}}{{\mathop \sum \nolimits_{i = 1}^{M} \mathop \sum \nolimits_{j = 1}^{N} [w_{X} \left( {i,j} \right) + w_{Y} \left( {i,j} \right)]}}$$

where, $$q\left( {i,j} \right) = \left\{ {\begin{array}{ll} {1,} & {if \quad s_{F} \left( {i,j} \right) < s_{X} \left( {i,j} \right) or \quad s_{F} \left( {i,j} \right) < s_{Y} \left( {i,j} \right)} \\ {0,} & { else } \\ \end{array} } \right.$$

#### Entropy

Entropy $(H _{XY}^{F} )$ (Haghighat et al. 2011) of an image is an important statistical parameter used to measure the quantity of information contained in it. The value of entropy depicts the amount of information present in the image. Mathematically,

17 $$H _{XY}^{F} = - \mathop \sum \limits_{k = 0}^{L - 1} p_{k} log_{2} \left( {p_{k} } \right)$$

where L is the number of gray levels in an image and *p*_*k*_ is the probability associated with *k*th gray level of image F.

#### Standard deviation

Standard deviation (*S*_*XY*_^*F*^) (Haghighat et al. 2011) is defined as the square root of variance and is used to determine the details contained in an image by measuring the contrast level present in it. The large value of standard deviation means that the image has higher degree of clarity and contrast. Mathematically,

18 $$S_{XY}^{F} = \sqrt {\frac{{\mathop \sum \nolimits_{i = 1}^{M} \mathop \sum \nolimits_{j = 1}^{N} (f(i,j) - \mu )^{2} }}{M \times N}}$$

where $f (i, j)$ is the intensity of pixel in *i*th row and *j*th column and $\mu$ is the mean of the image F.

#### Feature mutual information

Feature mutual information ($FMI_{XY}^{F}$) metric proposed by Haghighat (Arathi and Soman 2009) calculates the amount of feature information, FI_XF_ and FI_YF_ transferred from X and Y into F respectively. Mathematically,

19 $$FMI_{XY}^{F} = FI_{XF} + FI_{YF}$$

where, $FI_{XF} = \mathop \sum \nolimits_{F,X} p_{FX} (i,j,k,l)log_{2} \frac{{p_{FX} (i,j,k,l)}}{{p_{F} (i,j)p_{X} (k,l)}}$ and $FI_{YF} = \mathop \sum \nolimits_{F,Y} p_{FY} (i,j,k,l)log_{2} \frac{{p_{FY} (i,j,k,l)}}{{p_{F} (i,j)p_{Y} (k,l)}}$ where $p_{FX}$ ($p_{FY}$) are the joint distribution function between $X (Y)$ and $F$.

#### Fusion factor

Fusion factor ($FF_{XY}^{F}$) (Wang et al. 2004) determines the amount of mutual information between each individual input image and the fused image. The large value of $FF_{XY}^{F}$ means that good enough amount of information has been transferred from the source images to the fused image. Mathematically,

20 $$FF_{XY}^{F} = MI_{XF} + MI_{YF}$$

where $MI_{XF} (MI_{YF} )$ are the mutual information between $X (Y)$ and F.

#### Fusion symmetry

The parameter fusion symmetry ($FS_{XY}^{F}$) (Wang et al. 2004) was introduced to indicate the symmetry of the process with respect to the input images. Smaller the value of $FS_{XY}^{F}$, better is the performance of the fusion process. Mathematically,

21 $$FS_{XY}^{F} = \frac{{(MI_{XF} - MI_{YF} )}}{{2(MI_{XF} + MI_{YF} )}}$$

#### Structural similarity index measure

Structural similarity index measure ($SSIM_{XY}^{F}$) (Zhang et al. 2011) is used for determining the structural similarity between two images as it takes into account the characteristics of human visual system. The $SSIM_{XY}^{F}$ of the image X and F is given as

22 $$SSIM_{XY}^{F} = \frac{{\mathop \sum \nolimits_{j = 1}^{W} SSIM(x_{j, } f_{j} )}}{W}$$

where W is the total number of windows chosen in the image. $SSIM(x_{j, } f_{j} )$ is the similarity of the image in the *j*th local window xj and f_j_ and is given by,

23 $$SSIM\left( {x_{j, } f_{j} } \right) = \frac{{(2\mu_{{x_{j} }} \mu_{{f_{j} }} + k_{1}^{2} L^{2} )(2\sigma_{{x_{j} }} \mu_{{f_{j} }} + k_{2}^{2} L^{2} )}}{{(\mu_{{x_{j} }}^{2} + \mu_{{f_{j} }}^{2} + k_{1}^{2} L^{2} )(\sigma_{{x_{j} }}^{2} + \sigma_{{f_{j} }}^{2} + k_{2}^{2} L^{2} )}}$$

where L is the dynamic range of pixel values, *μ*_*x*_ and $\mu_{f}$ are the local means, *σ*_*x*_^2^ and $\sigma_{f}^{2}$ are the variance and *σ*_*xf*_^2^ is the cross-covariance for windows, x and f respectively. Similarly, *SSIM*_*XY*_ of Y and F can be obtained. The overall structural similarity index measure of the image X, Y and F is then defined as:

24 $$SSIM_{XY}^{F} = avg(SSIM_{XF,} SSIM_{YF} )$$

#### Feature similarity index measure

Feature similarity index measure (*FSIM*_*XY*_^*F*^) proposed by Liu and Laganiere (2007) measures the similarity between a pair of images based on the combination of phase congruency (PC) and gradient magnitude (GM). The former provides information about local structures in an image and the latter provides the contrast information. The feature similarity index is defined as:

25 $$FSIM_{XF} = \frac{{\mathop \sum \nolimits_{i = 1}^{M} \mathop \sum \nolimits_{j = 1}^{N} S_{XF} \left( {i,j} \right){ \hbox{max} }[PC_{X} \left( {i,j} \right),F\left( {i,j} \right) ]}}{{\mathop \sum \nolimits_{i = 1}^{M} \mathop \sum \nolimits_{j = 1}^{N} { \hbox{max} }[PC_{X} \left( {i,j} \right),F\left( {i,j} \right) ]}}$$

where *PC*_*X*_ and *PC*_*F*_ are the phase congruency values determined for X and F respectively. *S*_*XF*_(*i*, *j*) is the local similarity value. Similarly, the feature similarity index (*FSIM*_*YF*_) for grayscale images Y and F can be obtained. The overall feature similarity index is then defined as:

26 $$FSIM_{XY}^{F} = avg(FSIM_{XF} , FSIM_{YF} )$$

**Fig. 6:**
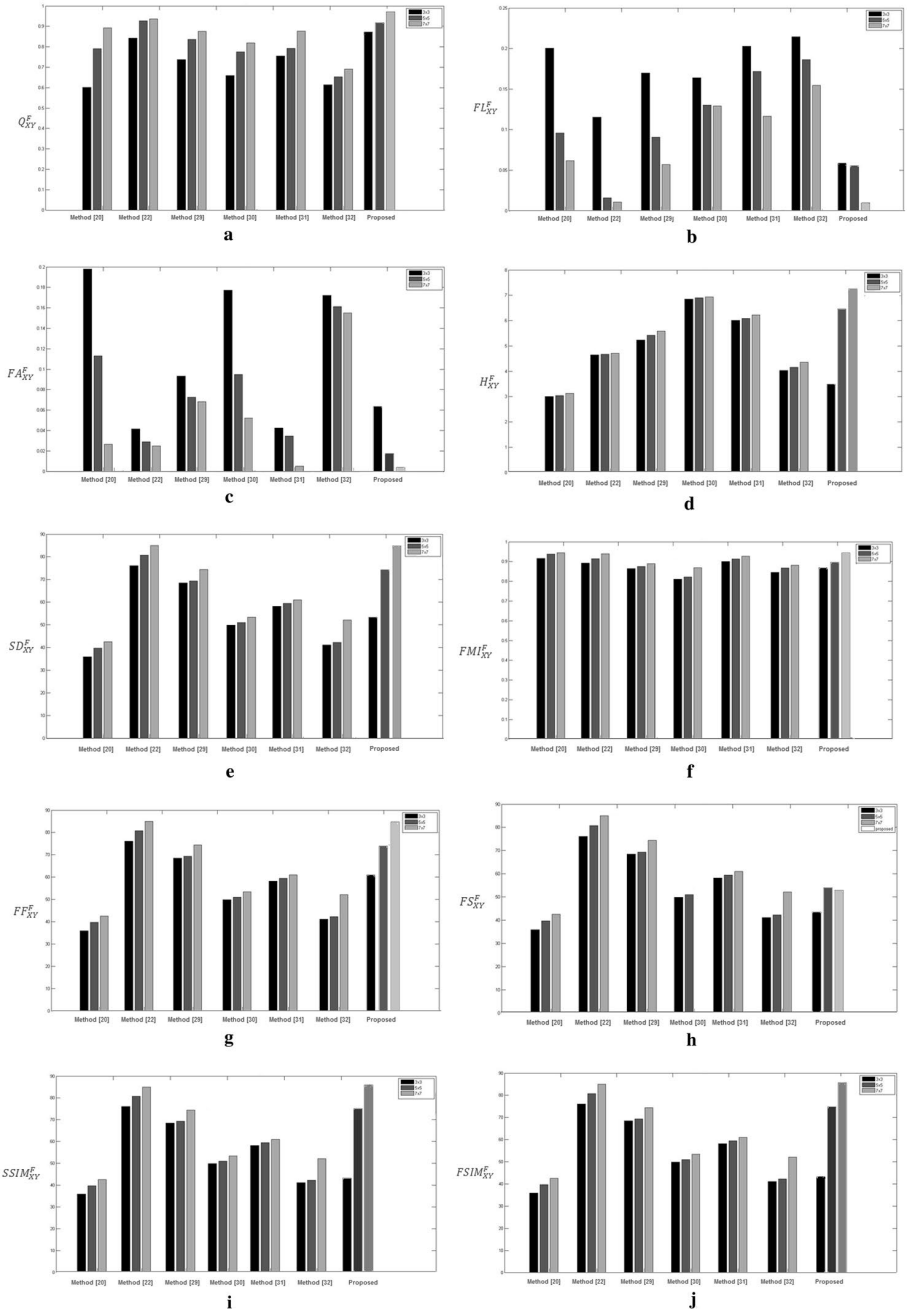
Quantitative comparison of various fusion methods.** a** Edge strength (*Q*_*XY*_^*F*^),** b** Fusion loss (*FL* ^*XY*^ ^*F*)^,** c** Fusion artifacts* FA*_*XY*_^*F*^,** d** Entropy (*H*_*XY*_^*F*^),** e** Standard deviation (*S*_*XY*_^*F*^),** f** Feature mutual information (*FMI*_*XY*_^*F*^), **g** Fusion Factor (*FF*_*XY*_^*F*^),** h** Fusion symmetry (*FS*_*XY*_^*F*^), **i** Structural similarity index measure (*SSIM*_*XY*_^*F*^ ), **j** Feature similarity index measure (*FSIM*_*XY*_^*F*^)

Values of various objective measures obtained using different methods are compared in Fig. 6. From observing these values, it is clear that high values of edge strength, entropy, standard deviation, feature mutual information, fusion factor, structural symmetry index measure and feature similarity index measure and low values of fusion loss, fusion artifacts and fusion symmetry imply a better quality of fused image. The contrast information of the source images is emphasized and enhanced in the proposed method. Consequently, the fusion rules of proposed algorithm based on maximum directional contrast can enhance the contrast, local characteristic and details from source images. Figure 6a, d compares edge strength and entropy metric. It is observed that using proposed method edge information is preserved and it has less contrast distortions. On the other hand, the existing methods like (Kumar 2013; Singh and Khare 2014; Wang et al. 2013; Bhatnagar et al. 2013) use the concept of wavelet decomposition to obtain wavelet coefficients, but they have lower value of entropy of fused image as compared to proposed method. This is due to the fact that, for wavelet decomposition of the source images, a proper selection of mother wavelet is vital, otherwise distortion and noise is observed in output image. For our proposed method as higher valued wavelet coefficients carry salient information about images such as edges, and corners, therefore, we have chosen maximum selection rule for fusion. Figure 6b compares fusion loss. It is observed that method (Kumar 2013) shows some comparable results, but in this method the loss is incurred due to sparse relationship where directional details are concerned rather than fine. Figure 6c compares fusion artifacts. Kumar (2013), Wang et al. (2014) shows comparable results with the proposed method. In SIST method cross-scale and inter sub-bands have been fully considered in the fusion rule but maximum and minimum fusion rules are not considered. This means if noise is stronger at any corner or edge it will get weighted summation which degrades the image quality. When other wavelet based methods are considered, low-frequency coefficients are fused by the averaging method, meaning the fused coefficients are the average of the corresponding coefficients of the source images. The high-frequency coefficients are fused by choosing absolute maximum. Figure 6e compares standard deviation. The results from DCHWT method is comparable to proposed method, but this method has shift variance problem at the cost of an over-complete signal representation. Figure 6f compares feature mutual information. It is observed that the larger absolute values of high-frequency coefficients correspond to the sharper brightness in the image and lead to the salient features such as edges, lines, region boundaries, and so on. However, these are very sensitive to the noise and therefore, the noise will be taken as the useful information and misinterpret the actual information in the fused images. To select high-frequency coefficients is necessary to ensure better information interpretation. Figure 6g, h compares fusion factor and fusion symmetry respectively. These are related to mutual information content of image. It is observed that proposed method shows overall better values. Figure 6i, j compares structural similarity index measure and feature similarity index measure. Some methods show similar performance in some cases and poor performance in other cases. From these results, it is concluded that the metrics obtain their best value using the proposed method. Thus, both subjective and objective comparison proves the superiority of proposed algorithm.

## Conclusion

This paper proposes image fusion method based on contrast based fusion rule in FTR domain. Capability of FTR in preserving monotonicity and Lipschitz continuity of a function helps in efficient reconstruction of fused image. Choice of directional contrast rule to fuse FTR components and select maximum based rule to fuse inverse-FTR components extracts all prominent information that is present in input images and provides more informative fused image. Results obtained from proposed algorithm set of images are visually as well as quantitatively compared with those obtained using other standard and recent methods. The fused image obtained using proposed method contains richer feature and detailed information than other fused images. Both visual and quantitative results prove the superiority of proposed algorithm.
